# Infant Oral Healthcare and Anticipatory Guidance Practices among Dentists in a Pediatric Care Shortage Area

**DOI:** 10.1155/2021/6645279

**Published:** 2021-03-23

**Authors:** Eman A. Bakhurji, Hassan M. Al-Saif, Mohammed A. Al-Shehri, Khalid M. Al-Ghamdi, Mohamed M. Hassan

**Affiliations:** ^1^Department of Preventive Dental Sciences, College of Dentistry, Imam Abdulrahman Bin Faisal University, Dammam, Saudi Arabia; ^2^Qilwah General Hospital, Al Baha, Saudi Arabia; ^3^College of Dentistry, Imam Abdulrahman Bin Faisal University, Dammam, Saudi Arabia; ^4^Department of Preventive Dental Services, College of Dentistry, King Faisal University, Al Baha, Saudi Arabia

## Abstract

**Objectives:**

To assess dentists' practices and barriers towards infant oral healthcare (IOH) and anticipatory guidance (AG) in eastern Saudi Arabia.

**Methods:**

A regional, cross-sectional survey was distributed to 340 (323 general dentists (GPs) and 17 pediatric dentists (PDs)) working in a governmental setting in eastern Saudi Arabia. A 23 close-ended, pilot-tested questionnaire was developed. The questionnaire asked about dentists' IOH and AG practices. A five-point Likert scale question assessed barriers interfering with AG practices. Descriptive and multivariate logistic regressions were used.

**Results:**

Participation rate was 98.5% (335/340). Only 18% of GPs indicated performing IOH exams, while 100% of PDs do. About 90% of GPs would see children on a first visit when they are ≥3 years old, whereas 60% of PDs reported seeing one-year-old children. Older practitioners and those performing AG were more likely to perform IOH (OR = 1.8, CI = 1.06–3.1, and OR = 3.84, CI = 1.93–7.65, resp.). The majority of respondents (94%) felt their training did not prepare them to practice AG. “Parents bringing their children for the first time for emergency or existing conditions” was cited by 99% of respondents as a barrier to performing AG.

**Conclusion:**

Increasing the awareness of GPs and parents about the importance of IOH and AG is crucial in improving children's oral health. Collaboration with pediatricians for early referral of children is equally important in increasing the awareness on prevention principles.

## 1. Introduction

The American Academy of Pediatric Dentistry (AAPD), three decades ago, has established guidelines for infant oral healthcare that are specifically directed towards young children. Infant oral healthcare aims at delivering preventive dental strategies within six months after the eruption of the first primary tooth and no later than 12 months of age [1]. Having this initial dental visit helps establish a dental home for the child at an early stage of life to prevent common dental diseases [2]. The guidelines recommend that oral healthcare providers use a proactive approach for evaluation of caries risk that utilizes an anticipatory guidance method [1]. Anticipatory guidance (AG) is an effective, prospective, chronologically based counselling approach, which educates parents about the upcoming dental needs at different developmental phases [3, 4]. Unlike the traditional educational method where the doctor provides information and the parent listens, AG provides concise, less static massage and interaction between the dentist and parents [1]. Dentists are encouraged to have one-on-one educational sessions with the parents to talk about their child's dental health and provide information about teething, sealants, and exfoliation. Alternatively, parents can ask questions which help them understand their child's dental development and prevent anticipated problems [5, 6].

Early childhood caries (ECC) is a special form of dental caries characterized by rapid progression on smooth tooth surfaces soon after eruption and has adverse effects on the dentition and quality of the child's life [7, 8]. Early dental caries is known as a risk factor for future caries, where ECC increases caries incidence up to 17-fold later in life [9]. Although there is a significant decrease in the incidence of caries in permanent teeth, the incidence of deciduous teeth caries is still growing [10, 11]. This could be attributed to the very rapid progression of caries in the primary teeth in a six-to-twelve-month period, which requires immediate and early dental intervention to address risk factors that lead to ECC [10]. Risk factors associated with ECC could include the introduction of unfavorable dietary habits early in the child's life such as bed bottle feeding and using a nonspill cup and sweetened pacifiers. In older children, ECC could be attributed to frequent intake of sugary snacks and drinks [11]. In addition to diet, insufficient oral hygiene practices and low exposure to fluorides and lack of parental knowledge are also associated with the development of ECC [12]. Therefore, early prevention could decrease the child's risk to develop caries later in life.

Saudi Arabia is in no better situation when it comes to dental caries [11, 12]. In the eastern province, the overall prevalence of dental caries in children is also very high (73%) [13]. The high prevalence of dental caries in the region and lack of public and parental awareness [14, 15] call for increasing the efforts to educate parents and provide early intervention based on the AAPD infants oral healthcare guidelines utilizing anticipatory guidance concepts and establishing a dental home at an early age of life. Previous studies in the US suggest a lack of dentists' compliance, especially general dentists, with the AAPD guidelines although most of them believed in early intervention [16, 17]. However, it is unknown if dentists in the area are aware of the AAPD guidelines or seeing children at an early age. Therefore, the aims of this study are to evaluate the practices of general and pediatric dentists in the eastern province, Saudi Arabia, towards the implementation of anticipatory guidance and infant oral health visit and to assess the barriers facing them that interfere with these practices.

## 2. Materials and Methods

This regional, cross-sectional study targeted general and pediatric dentists working in governmental settings in the eastern province of Saudi Arabia. The list of potential participants was provided by the Saudi Commission of Health Specialists who provide dental licensures to dentists in Saudi Arabia. According to the Saudi Commission of Health Specialists, there are only 17 licensed and registered pediatric dentists in the eastern province and 323 general practitioners working in governmental hospitals and health centers. A total of 340 pediatric and general dentists were approached to participate in this study.

Data collection included a self-administered questionnaire that was developed in English and Arabic based on previous studies. [16, 18, 19]. The questionnaire included 23 close-ended questions divided in three sections. Section A included four questions that investigated demographical and work-related variables such as gender, academic background, and practice patterns. Section B had 18 questions that investigated the current practices and attitudes of dentists regarding AG. Section C had one question that used a five-point Likert scale (ranging from strongly agree to strongly disagree) to assess barriers that prevent dentists from implementing anticipatory guidance. The questionnaire had a cover letter that explained the purpose and significance of the study along with a definition of anticipatory guidance to ensure consistent understanding among participants.

To ensure clarity and validity of the questions, the questionnaire was reviewed and pilot-tested by a dental public health survey expert who was not involved in the study. Ethical approval was obtained from Institutional Review Board at College of Dentistry, Imam Abdulrahman Bin Faisal University. To distribute the survey to general dentists, the research team visited hospitals and healthcare centers to manually administer the survey. However, the survey was distributed to pediatric dentists during one of their monthly club meetings. Involvement in the study was voluntary and imposed a minimal risk. Respondents were not compensated for their participation in the survey.

The Statistical Package of Social Science (SPSS) Version 24 was used for descriptive and multivariate logistics analysis. The primary outcome was performing infant oral healthcare exams. Categorical variables were presented in frequencies and percentages for demographical, attitude, and practices questions. The barrier question that used a five-point Likert scale was recategorized into three groups (agree, neutral, and disagree) for presentation. Variables included in the multivariate model are those who were statistically significant in the bivariate analysis. Significance level was set at 5%.

## 3. Results

### 3.1. Response Rate and Demographical Distribution

Of the 340 surveys distributed, 335 were returned for an overall participation rate of 98.5%. The response rate for general dentists was (318/323) 98.5%, while it was (17/17) 100% for pediatric dentists. More than two thirds of the participants were males (76%), and most of them obtained their highest degree from a program in Saudi Arabia (86%) (data not tabulated).

### 3.2. Dentists Practices regarding Infant Oral Healthcare and Anticipatory Guidance

**Figure 1:**
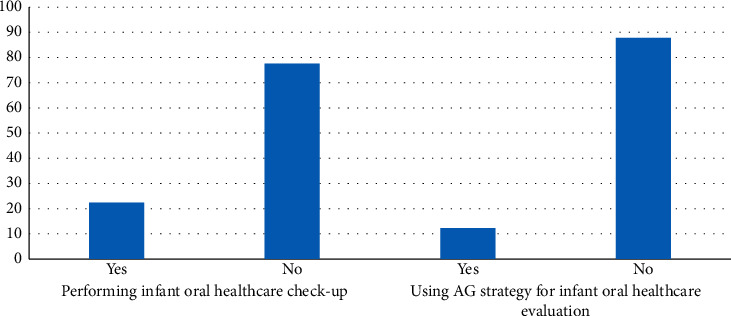
Distribution of dentists' attitude towards infant oral healthcare and AG (*N* = 335).

**Figure 2:**
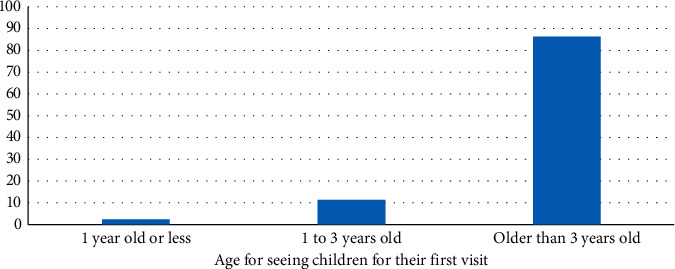
Distribution of age for children's first dental visit (*N* = 335).

Figure 1 shows that the majority of responding dentists do not routinely perform infant oral health examinations in their offices (78%), and approximately 88% of the participants do not use an anticipatory guidance (AG) strategy in their practices. Particularly, only 18% of general dentists do perform infant oral healthcare examination or perform AG. Meanwhile, 100% of responding pediatric dentists indicated that they perform oral health examination and perform AG in their offices. All participants responded that they routinely evaluate children's oral development, examine for oral pathology and dental decay, give oral hygiene instructions, and educate parents about dental decay (100%). More than half of the participants indicated that they do nutritional counselling and discuss the risk of baby bottle decay (64% of general dentists and 60% of pediatric dentists). However, as seen in Figure 2, the majority of participants indicated that they see children in their practice for the first time when they are older than three years old (86.3%), and they were mostly general dentists (91% of general dentists). Alternatively, only 2.4% of respondents indicated that they see children by the age of one, and they are more likely to be pediatric dentists (61% of pediatric dentists).

### 3.3. Dentists Practices of AG

Table 1 shows that all participants who indicated performing anticipatory guidance in their practice reported having a standard strategy for conducting AG in their offices (100%). More than half of the participants who have a strategy to perform AG (58.5%) reported that they let a staff member deliver AG and 62.5% of those staff members have been formally trained to conduct AG. Most of the trained staff were dental assistants (80%). The majority of the participants who had been trained to perform AG got their training during postgraduate programs (80%), while only 20% indicated that they got their training through continuous education courses (CE). Two thirds of those performing AG felt that their training was insufficient (66.7%).

### 3.4. Barriers Interfering with Performing AG

Table 2 shows that 54% of the participants disagreed that “parents not seeing the value of AG counselling” is a barrier to performing AG. Two thirds of the respondents (65.7%) agreed that being too busy treating older patients prevented them from performing AG counselling. More than half (54%) of the participants agreed that AG is time consuming, while 38.2% disagreed. However, 61% of responding dentists were not decisive if AG is beneficial to perform. Most dentists (94.3%) agreed that they are not trained enough to perform AG. The most cited potential barrier respondents (99.4%) agreed with was that parents bring their children for the first time to a dentist only for emergency management or to address existing conditions.

### 3.5. Factors Associated with Infant Oral Healthcare Practices

Table 3 shows results from the multivariate logistic regression model. Surprisingly, dentists who graduated before 2010 were more likely to perform infant oral healthcare exams than those who graduated in 2010 or later (OR = 1.8, CI = 1.06–3.1). Additionally, those who performed anticipatory guidance were about four times more likely to perform infant oral healthcare exams (CI = 1.93–7.65).

## 4. Discussion

This study conducted a regional survey among dentists practicing in a governmental setting in the eastern province of Saudi Arabia to investigate their practices about infant oral healthcare and anticipatory guidance. These are important preventive strategies directed towards young children, and they are particularly important in the Middle East where dental caries is a significant dental and public health issue affecting most children while suffering from a lack of specialized workforce. Our research found that most of the general dentists included in this study do not perform infant oral healthcare exams or use AG strategies or see very young children in their offices. “Parents bringing their children to the dentist only for emergencies or to address existing conditions” was one of the major barriers that interfered with dentists' practice of infant oral healthcare exams and AG.

Although all pediatric dentists involved in this study reported performing infant oral health evaluations and indicated that they perform anticipatory guidance counselling during the visit, most general dentists do not. Similarly, the majority of pediatric dentists would see children by the age of one, while most general dentists would see children when they are three years old or older. These findings are consistent with previous studies which indicated that very few general dentists perform infant oral healthcare exams or prefer to see very young children [16, 17, 20, 21]. General dentists may not be comfortable seeing very young children because of the lack of appropriate undergraduate training to perform preventive services for young children. This is also consistent with our findings, where almost all respondents agreed that their dental training did not prepare them well to perform AG. Additionally, our study found that most dentists got their AG training during their postgraduate education or through continuous education courses. This raises the concern of why participants felt that their undergraduate programs do not prepare them well to perform anticipatory guidance although infant oral health and anticipatory guidance counselling are supposed to be part of the dental curriculum. However, even in the US, dental schools dedicate an average of only two hours of its curriculum on infant oral healthcare, while 50% of dental schools provide clinical training for treating the infant population [22]. Dental students were found more comfortable treating young children following an infant oral health care educational program that involved four clinical sessions [23]. In a similar study conducted in a Saudi dental school, a 14 min PowerPoint educational session on infant oral healthcare was found effective in improving the knowledge of female students [24]. Therefore, expanding the educational efforts to incorporate the principles of infant oral healthcare, dental home, and AG in the undergraduate dental curricula is promising and required. This recommendation becomes essential in the eastern area of Saudi Arabia, where very few dentists are specialized in pediatric dentistry and general dentists must see most of the pediatric population.

While all pediatric dentists in this study reported performing infant oral healthcare exams, a lower percentage indicated seeing children by the age of one. Malcheff et al. and Brickhouse et al. reported similar results [16, 20]. Most studies referred this low compliance with the AAPD guidelines to the fact that these preventive services are not cost-effective compared with restorative care under general anesthesia and sedation. However, since our study included dentists practicing only in a governmental setting where services are provided for free, financial reasons do not explain the low compliance. Instead, it could be explained by parents' lack of awareness about the importance of the one-year-old visit. This explanation is consistent with our finding where almost all respondents agreed that parents bring their children to the dentists only for an emergency and more than half of the respondents disagreed that parents do not see the value of AG. This finding indicates that dentists perceive parents as oral health advocates and supportive of anticipatory guidance counselling. They play a major role in the dynamics of delivering preventive services and their education could shift the paradigm of dental caries and oral diseases.

Early parental education focuses on identifying risk factors such as dietary habits, fluoride exposure, and plaque accumulation [25]. Parents should learn about the risk of developing ECC caused by night bottle feeding, ad libitum breastfeeding, use of sippy cups, and increased consumption of sugars between meals [26, 27]. The prevalence of consumption of sweetened-sugar beverages among toddlers and young children is increasing and many parents are introducing 100% fruit juice to their infants before their first birthday [28]. Rusali et al. found that children who were bottle-fed in bed or were bottle-fed for up to four years and those who were weaned from the bottle by introducing sweet drinks are significantly at a higher risk for developing ECC [29]. These improper parental behaviors increase their children risk for ECC early in life and that in turn shifts the dental practice from being prevention-driven to restoration-driven.

Another potential barrier to performing infant oral healthcare exams and AG is that pediatricians are not encouraging parents to take their children to see the dentist by 12 months of age. Although the American Academy of Pediatrics (AAP) focuses on the establishment of the dental home by age one in their policies, only 5% of US pediatricians recommended first dental visit by the age of one, while 69% recommended first dental visit at the age of three [16, 30]. Moreover, infants and young children have a greater access to pediatricians where they see a well-child 10 times before the age of three [2, 31]. This huge gap between medical and dental providers could create a delay in providing dental services, specifically prevention. As a result, infants and young children do not get the dental attention they require at an early age. Because pediatricians are the primary healthcare providers that pediatric patients first encounter and they can refer patients to the dentist, it would be useful to educate pediatricians about the importance of establishing a dental home by age one.

A limitation of our study is the inclusion of dentists working in governmental settings only. Further studies should include those working in private practice and investigate if financial considerations play a role in the provision of preventive services and early intervention. Due to the limited number of pediatric dentists in the area who are included in the study, we were not able to draw any statistical comparison between general and pediatric dentists. Additionally, findings from this study were based on dentists' reports instead of reviewing patients' records. However, due to the low compliance reported in the study, respondents' bias and overestimation of positive behavior are unlikely.

## 5. Conclusion

This study reported low compliance of general dentists with the AAPD guidelines for infant oral healthcare exams and AG. Dentists felt their undergraduate training was insufficient and most of the training they received was either during postgraduate training or through continuous education courses. “Parents bringing their children to the dentist for the first time to treat only emergency conditions” was the major barrier that interfered with the dentists' delivery of AG and early establishment of a dental home. The education of parents and pediatricians is crucial in increasing the awareness of the public and engaging them in the responsibility of early dental intervention. Dental schools should incorporate the principles of infant oral healthcare, AG, and early dental home in their curricula to enable graduating general dentists practice these concepts comfortably and efficiently.

## Figures and Tables

**Table 1:** Distribution of dentists' practices of AG.

AG practices		Frequency (%)
Have a standard strategy for conducting AG	Yes	41 (100%)
	No	0 (0%)

Other staff members involved in the delivery of AG	Yes	24 (58.5%)
	No	17 (41.5%)

Conduct formal staff training to conduct AG	Yes	15 (62.5%)
	No	9 (37.5%)

Staff members have you trained to conduct AG	Dentists	3 (20%)
	Assistants	12 (80%)

Got training to perform anticipatory guidance	Yes	15 (100%)
	No	0 (0%)

Time of AG training	Undergrads	0 (0%)
	Postgrads	12 (80%)
	CE	3 (20%)

The training was sufficient	Yes	5 (33.3%)
	No	10 (66.7%)

**Table 2:** Distribution of barriers interfering with practices of AG (*N* = 335).

	Disagree/strongly disagree *N* (%)	Neutral *N* (%)	Agree/strongly agree *N* (%)
Parents do not see the value of AG counselling	181 (54%)	133 (39.7%)	21 (6.3%)
Parents bring their children for the first time to a dentist only for emergency management or addressing existing conditions	1 (0.3%)	1 (0.3%)	333 (99.4%)
I am too busy with older patients to do AG counselling	40 (11.9%)	75 (22.4%)	220 (65.7%)
AG is time consuming	128 (38.2%)	26 (7.8%)	181 (54%)
I am not sure AG is beneficial	124 (37%)	204 (60.9%)	7 (2.1%)
I am not trained enough to perform AG	18 (5.4%)	1 (0.3%)	316 (94.3%)

**Table 3:** Multivariate logistic regression predicting IOH practices (*N* = 335).

Variable	OR	95% CI
*Year of graduation*		
<2010	1.815	1.06–3.10
≥2010 (ref)	—	—

*AG*		
Yes	3.84	1.93–7.65
No (ref)	—	—

## Data Availability

The data are available upon request from the corresponding author.
